# Methylation of *DACT2* accelerates esophageal cancer development by activating Wnt signaling

**DOI:** 10.18632/oncotarget.7647

**Published:** 2016-02-23

**Authors:** Meiying Zhang, Enqiang Linghu, Qimin Zhan, Tao He, Baoping Cao, Malcolm V. Brock, James G. Herman, Rong Xiang, Mingzhou Guo

**Affiliations:** ^1^ Department of Gastroenterology and Hepatology, Chinese PLA General Hospital, Beijing 100853, China; ^2^ Medical College of NanKai University, Tianjin 300071, China; ^3^ State Key Laboratory of Molecular Oncology, Cancer Institute and Hospital, Chinese Academy of Medical Sciences and Peking Union Medical College, Beijing 100021, P.R.China; ^4^ Sidney Kimmel Comprehensive Cancer Center, Johns Hopkins University, Baltimore, Maryland 21231, USA; ^5^ The Hillman Cancer Center, University of Pittsburgh Cancer Institute, Pittsburgh, PA 15213, USA

**Keywords:** DACT2, esophageal cancer, methylation, metastasis, wnt signaling

## Abstract

Esophageal cancer is one of the most common malignancies worldwide. *DACT2* is frequently methylated in human lung, hepatic, gastric and thyroid cancers. The methylation status and function of *DACT2* remain to be elucidated in human esophageal cancer. Ten esophageal cancer cell lines, 42 cases of dysplasia and 126 cases of primary esophageal cancer samples were analyzed in this study. The expression of DACT2 was detected in YES2 cells, while reduced DACT2 expression levels were found in TE8 and KYSE70 cells, and complete loss of DACT2 expression was found in KYSE30, KYSE140, KYSE150, KYSE410, KYSE450, TE3 and TE7 cells. Loss of expression or reduced expression of DACT2 correlated with promoter region hypermethylation in esophageal cancer cells. Restoration of DACT2 expression was induced by 5-aza-2′-deoxycytidine. In human primary esophageal squamous carcinoma, 69% (87/126) of samples were methylated. Methylation of *DACT2* was significantly associated with tumor stage and metastasis (*P* < 0.01, *P* < 0.05). DACT2 suppressed colony formation, cell migration and invasion in esophageal cancer cells, and it also suppressed esophageal cancer cell xenograft growth. DACT2 inhibited Wnt signaling in human esophageal cancer cells. In conclusion, *DACT2* is frequently methylated in human esophageal cancer and its expression is regulated by promoter region methylation. DACT2 suppresses esophageal cancer growth by inhibiting Wnt signaling.

## INTRODUCTION

Esophageal cancer is the sixth leading cause of cancer-related mortality and the eighth most common cancer worldwide. The 5-year overall survival ranges from 15% to 25% [1]. Squamous cell carcinoma is the predominant histological type of esophageal carcinoma in the world. The incidence of esophageal carcinoma varies widely by region [2]. In northern and central China, the incidence of esophageal Squamous cell carcinoma (ESCC) is more than 100 cases per 100,000 population annually [3]. In the past three decades, the incidence of esophageal SCC has declined, while the incidence of esophageal adenocarcinoma has been progressively increasing [4]. Tobacco and alcohol consumption are risk factors for ESCC. The combination of tobacco and alcohol consumption further increases the risk of SCC [5]. Gastro-esophagus reflux disease (GERD), obesity, Barrett's esophagus, and tobacco use are risk factors for esophageal adenocarcinoma. Nitrosamine exposure is a risk factor for both esophageal squamous carcinoma and adenocarcinoma. Risk has been demonstrated to decrease in patients with a history of *Helicobacter pylori* infection [6–9]. Both genetic and epigenetic changes are involved in the development of esophageal cancer [10–12].

The Wnt signaling pathway is reported to be involved in many phases of vertebrate embryonic development and the initiation and progression of human esophageal cancer [13–15]. Activation of the Wnt signaling pathway may cause accumulation of β-catenin in the cytoplasm and leads to its further translocation into the nucleus to regulate the downstream genes [16, 17]. Dapper is a Dishevelled-associated antagonist of β-catenin (DACT). It was identified by screening proteins that interacted with Dishevelled, a key factor in the Wnt signaling pathway [18]. Human *DACT2* was identified by Katoh et al., and it is located on human chromosome 6q27 [19]. *DACT2* silencing by promoter region hypermethylation was detected in many tumor types including lung, gastric, hepatocellular and thyroid cancers [20–23]. The epigenetic changes and functions of *DACT2* in human esophageal cancer remain to be elucidated. Therefore, in this study, we analyzed the epigenetic changes and functions of *DACT2* in human esophageal ESCC.

## RESULTS

### The expression of DACT2 is down-regulated by promoter region hypermethylation in human ESCC

**Figure 1:**
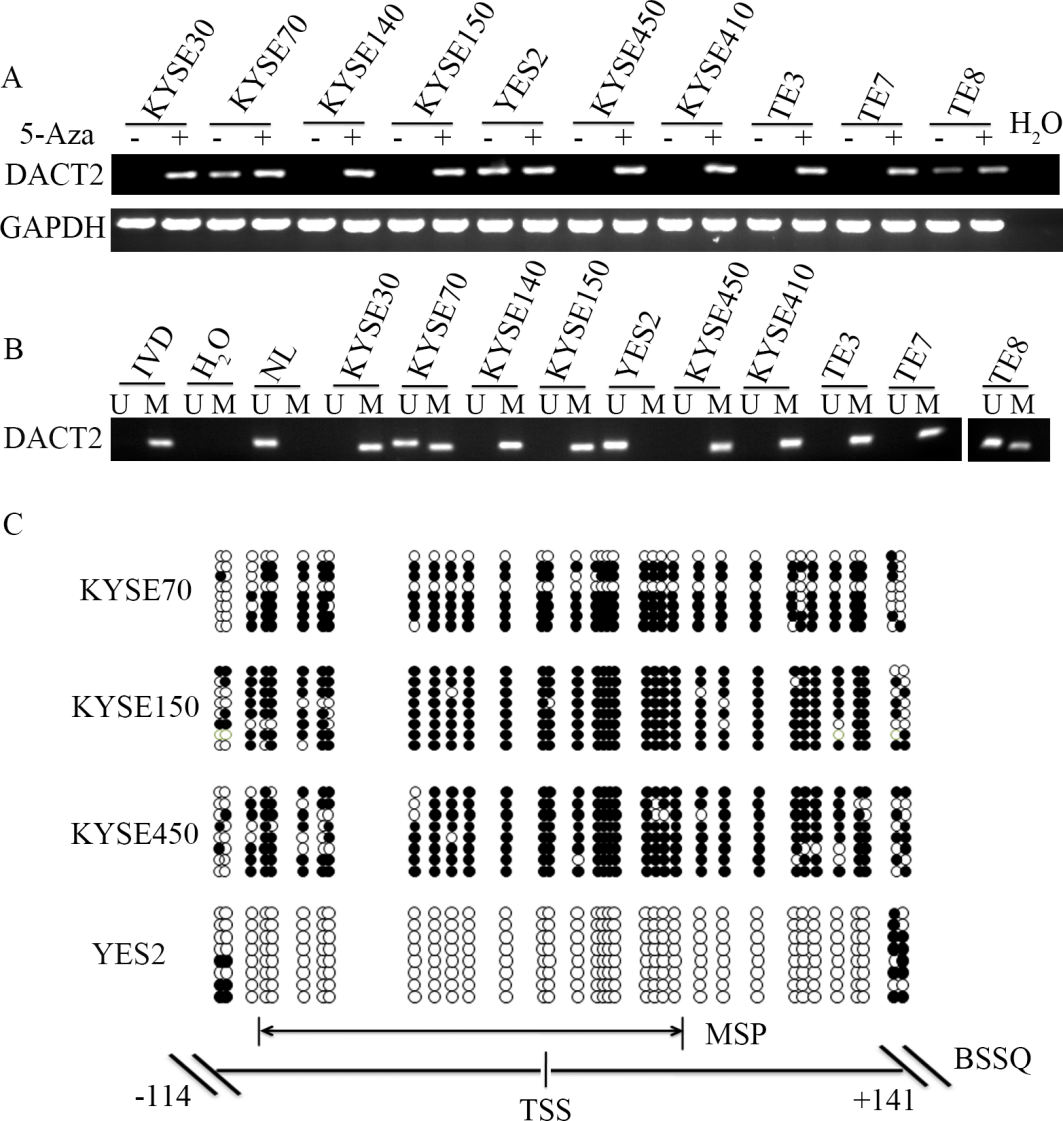
The expression of DACT2 is regulated by promoter region methylation in esophageal cancer cell lines (**A**) The expression of DACT2 was detected by semi-quantitative RT-PCR. H_2_O: negative control. GAPDH: internal control. 5-Aza: 5-aza-2′-dexycytidine; -: absence of 5-Aza; +: presence of 5-Aza. (**B**) MSP results in esophageal cancer cell lines. IVD: *in vitro* methylated DNA (methylation control); NL: lymphocyte DNA (unmethylation control); U: unmethylated alleles; M: methylated alleles. (**C**) Bisulfite Sequencing results: Double-headed arrow indicates the region of the MSP product. Filled circles: methylated CpG sites; open circles: unmethylated CpG sites. TSS: transcription start site.

The expression of DACT2 was detected by semi-quantitative RT-PCR in human esophageal cancer cells. DACT2 expression was detected in YES2 cells, while expression of DACT2 was reduced in TE8 and KYSE70 cells, and no expression was detected in KYSE30, KYSE140, KYSE150, KYSE410, KYSE450, TE3 and TE7 cells (Figure 1A). The promoter region methylation was examined by Methylation-Specific PCR (MSP). Complete methylation was found in KYSE30, KYSE140, KYSE150, KYSE410, KYSE450, TE3 and TE7 cells, and partial methylation was found in TE8 and KYSE70 cells. Unmethylation was found in YES2 cells (Figure 1B). These results indicated that loss or reduced expression of DACT2 is correlated with promoter hypermethylation in esophageal cancer cell lines. To further determine whether the expression of DACT2 was regulated by promoter region methylation, 5-aza-2′-dexycytidine (5-Aza) was used in this study. As expected, re-expression of DACT2 was found in KYSE30, KYSE140, KYSE150, KYSE410, KYSE450, TE3 and TE7 cells after 5-Aza treatment, and increased expression of DACT2 was detected in TE8 and KYSE70 cells (Figure 1A). These results demonstrated that DACT2 expression is regulated by promoter region methylation in human esophageal cancer cells.

To validate the efficiency of the MSP primers, bisulfite sequencing was employed. Dense methylation was observed in the promoter region of *DACT2* in KYSE150 and KYSE450 cells, while partial methylation was detected in KYSE70 cells and unmethylation was found in YES2 cells (Figure 1C). The above results further suggest that the expression of DACT2 is regulated by promoter region methylation.

### *DACT2* is frequently methylated in human primary esophageal cancer

**Figure 2:**
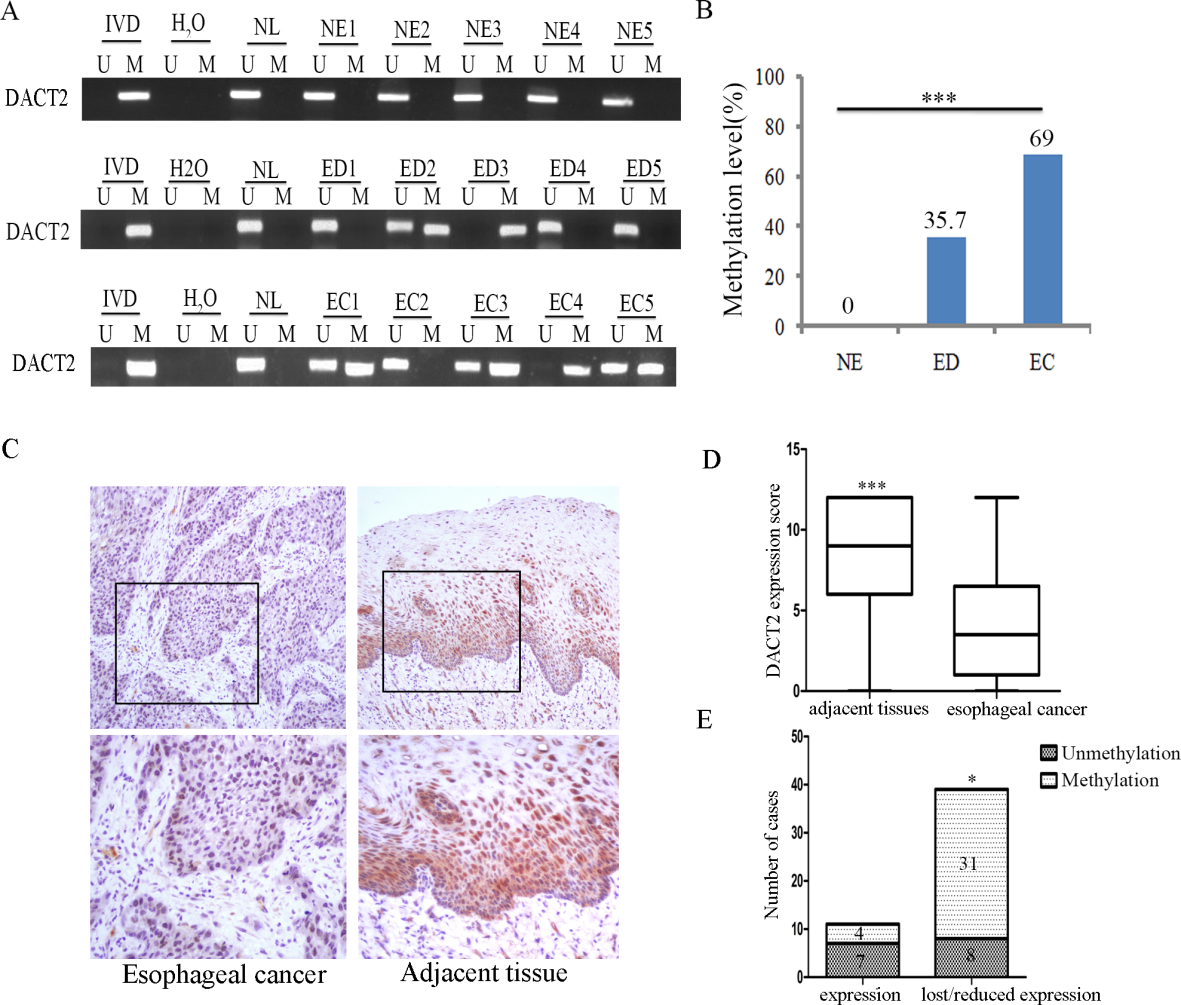
The methylation and expression status of DACT2 in primary esophageal cancer (**A**) Representative methylation results of *DACT2* in normal esophageal mucosa (NE), esophageal dysplasia (ED) and esophageal cancer (EC). (**B**) *DACT2* methylation frequency in NE, ED and EC. The frequency of *DACT2* methylation was analyzed by chi-square test. ***P < 0.001. (**C**) Representative IHC staining of DACT2 in esophageal cancer (left panels) and adjacent tissue (right panels). Upper panels: ×200; lower panels: ×400. (**D**) DACT2 expression scores are shown as box plots, horizontal lines represent the median score; the bottom and top of the boxes represent the 25th and 75th percentiles, respectively; vertical bars represent the range of data. Expression of DACT2 was different between adjacent tissue and esophageal cancer tissue in 50-matched primary esophageal cancer samples. ***P < 0.001. (**E**) The expression level of DACT2 and DNA methylation status is shown as a bar diagram. Reduced expression of DACT2 was significantly associated with promoter region hypermethylation. *P < 0.05.

The methylation status of *DACT2* was detected by MSP in 126 cases of primary ESCC, 42 cases of dysplasia and 27 cases of normal esophageal mucosa. 69% (87/126) of primary esophageal cancer samples, 35.7% (15/42) of dysplasia samples were methylated and no methylation (0/27) was found in normal esophageal mucosa. The frequency of *DACT2* methylation was increased in progression tendency during esophageal development (*P* < 0.001) (Figure 2A and 2B). Methylation of *DACT2* was significantly associated with tumor stage and lymph node metastasis (Table 1, *P* < 0.01, *P* < 0.05), while no association was found between *DACT2* methylation and gender, age, differentiation and tumor size (all *P* > 0.05). The expression of DACT2 was evaluated by immunohistochemistry in 50 cases of available matched esophageal ESCC and adjacent tissue samples. The staining intensity and extent of the stained area were scored using the German semi-quantitative scoring system. DACT2 staining was existed in both the nucleus and cytoplasm (Figure 2C). The expression level of DACT2 was significantly reduced in cancer tissue samples compared with adjacent tissue samples (Figure 2D, *P* < 0.001). Reduced expression was found in 39 cases of cancer tissue. Among the 39 cases in which reduced levels of DACT2 were detected, 31 cases were methylated. The reduced expression of DACT2 was significantly associated with promoter region hypermethylation (Figure 2E, *P* < 0.05). These results indicate that the expression of DACT2 is regulated by promoter region methylation in primary esophageal cancer.

**Table 1:** Clinic-pathological features and *DACT2* methylation status in esophageal cancer patients

Clinical parameter	NO.	Methylation status		*P* value*
		Methylated *n* = 87	Unmethylated *n* =39	
**Gender**				
Male	91	59	32	*p* = 0.099
Female	35	28	7	
**Age**				
≥ 60	81	56	25	*p* = 0.98
< 60	45	31	14	
**Differentiation**				
Moderately/Well	70	49	21	*p* = 0.796
Poorly	56	38	18	
**Tumor stage**				
I/II	83	65	18	*p* = 0.002**
III/IV	43	22	21	
**Metastasis**				
Positive	57	33	24	*p* = 0.014*
Negative	69	54	15	
**Tumor size**				
≥ 5 cm	51	33	18	*p* = 0.385
< 5 cm	75	54	21	

* *P* values are obtained from χ^2^ test, significant difference, *P < 0.05,

** *P* < 0.01.

### DACT2 suppresses esophageal cancer cell proliferation

**Figure 3:**
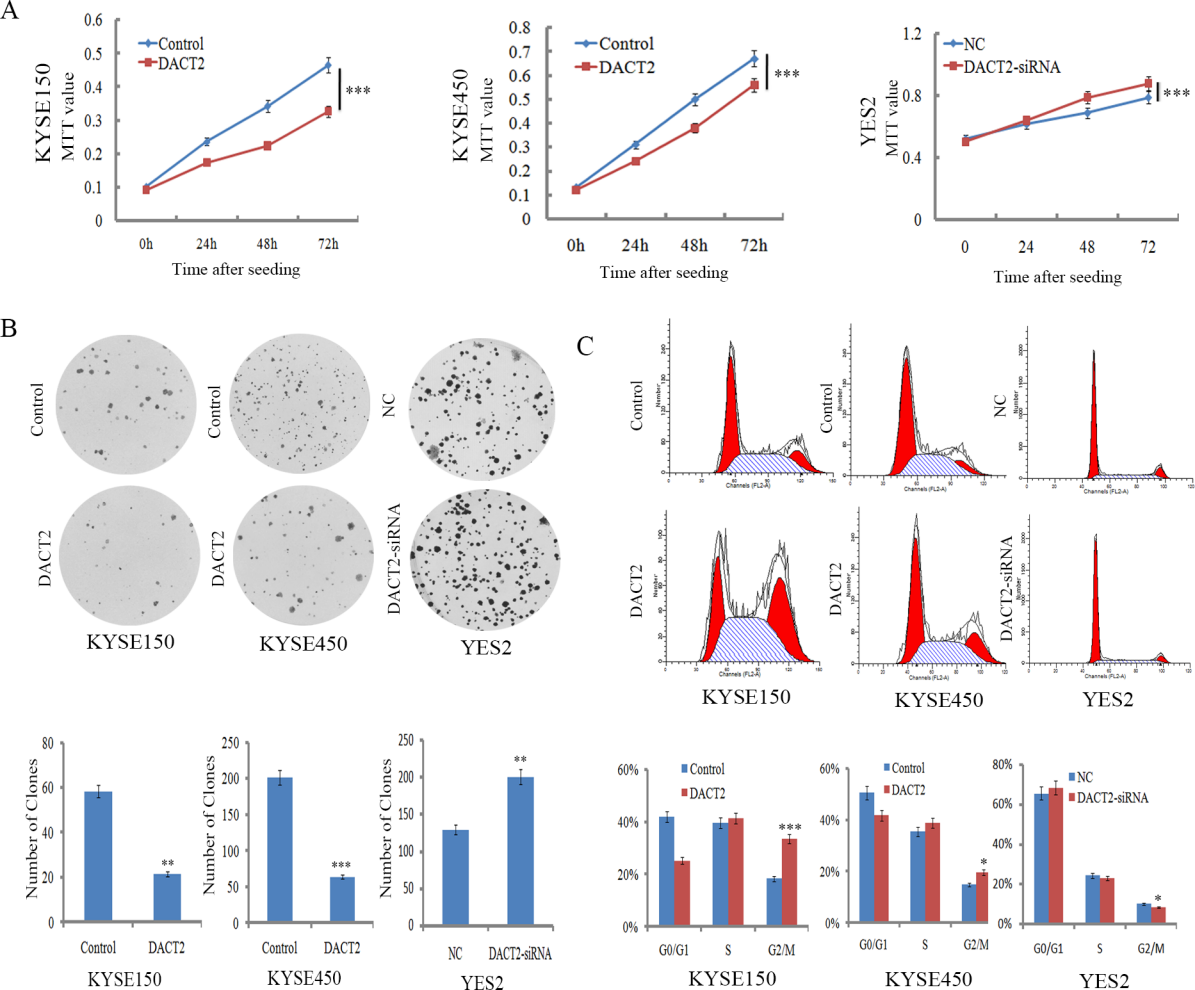
DACT2 inhibits cell proliferation and induces G2/M phase arrest in esophageal cancer cells (**A**) The effect of DACT2 on cell viability was measured by the MTT assay for 72 hours. Each experiment was repeated three times. ***P < 0.001. (**B**) Colony formation assays show that clone number is reduced after restoration of DACT2 expression in KYSE150 and KYSE450 cells, as well as colony formation results in YES2 cells before and after knockdown of DACT2. Each experiment was repeated three times. ***P < 0.001, **P < 0.01. (**C**) Cell phase distribution in DACT2 unexpressed and re-expressed KYSE150 and KYSE450 cells, as well as cell phase distribution before and after knockdown of DACT2 in YES2 cells. Each experiment was repeated three times. ***P < 0.001, *P < 0.05.

To evaluate the effect of DACT2 expression on cell growth, the MTT assay was employed. The OD values were 0.464 ± 0.024 *vs*. 0.326 ± 0.012 (*P* < 0.001) and 0.658 ± 0.037 *vs*. 0.56 ± 0.016 (*P* < 0.001) before and after restoration of DACT2 expression in KYSE150 cells and KYSE450 cells, respectively. Cell viability was reduced after restoration of DACT2 expression in KYSE150 and KYSE450 cells. The effect of DACT2 on cell growth was further validated by knocking down DACT2 in YES2 cells. The OD values were 0.787 ± 0.022 *vs*. 0.876 ± 0.033 (*P* < 0.001) before and after knockdown DACT2 in YES2 cells (Figure 3A). Cell viability was increased after knockdown of DACT2 in YES2 cells.

To evaluate the effect of DACT2 on clonogenicity in esophageal cancer, we performed a colony formation assay. The clone number was 58.3 ± 7.6 *vs*. 21.3 ± 4.2 (*P* < 0.01) in KYSE150 cells and 201.3 ± 24.2 *vs*. 63.7 ± 13.1 (*P* < 0.001) in KYSE450 cells before and after restoration of DACT2 expression. The number of clones was reduced after re-expression of DACT2 in KYSE150 and KYSE450 cell lines. The effect of DACT2 on clonogenicity was further validated by knocking down DACT2 in YES2 cells. The clone number was 129.3 ± 17.0 *vs*. 200.3 ± 4.5 (*P* < 0.01) before and after knockdown of DACT2 in YES2 cells (Figure 3B). These results suggest that DACT2 inhibits the proliferation of esophageal cancer cells.

### DACT2 induced G2/M phase arrest in esophageal cancer cells

To analyze the effect of DACT2 on the cell cycle, flow cytometry was employed. The distribution of cell phases in DACT2 unexpressed and re-expressed KYSE150 cell lines was 42.02 ± 1.96% *vs*. 25.21 ± 2.62% in G0/G1 phase, 39.70 ± 0.59% *vs*. 41.29 ± 1.74% in S phase, and 18.28 ± 1.38% *vs*. 33.49 ± 2.47% in G2/M phase. The G2/M phase was significantly different before and after re-expression of DACT2 in KYSE150 cells (*P* < 0.001). In KYSE450 cells, the cell phase distribution was 50.54 ± 1.65% *vs*. 41.76% ± 1.37% in G0/G1 phase, 35.37 ± 1.95% *vs*. 38.75 ± 2.47% in S phase, and 14.74 ± 1.42% *vs*. 19.59 ± 1.62% in G2/M phase before and after restoration of DACT2 expression. The G2/M phase was significantly different before and after re-expression of DACT2 in KYSE450 cells (*P* < 0.05). The effect of DACT2 on cell cycle was further validated by knocking down DACT2 in DACT2 highly expressed YES2 cells. The distribution of cell phases was 65.54 ± 1.96% *vs*. 68.49 ± 0.40% in G0/G1 phase, 24.33 ± 0.98% *vs*. 23.13 ± 0.17% in S phase, and 10.13 ± 0.97% *vs*. 8.37 ± 0.30% in G2/M phase. The G2/M phase was significantly reduced by knocking down DACT2 in YES2 cells (*P* < 0.05, Figure 3C). To further validate the effect of DACT2 on G2/M phase, the levels of cyclinB1, CDC2 and p-CDC2 (Y15) were examined by western blot in KYSE150 and KYSE450 cells before and after re-expression of DACT2. The expression levels of cyclinB1 and CDC2 were reduced, and the levels of p-CDC2 (Y15) were increased after re-expression of DACT2. The levels of cyclinB1, CDC2 and p-CDC2 (Y15) were examined as well before and after knockdown of DACT2 in YES2 cells. The levels of cyclinB1 and CDC2 were increased, and the levels of p-CDC (Y15) were reduced after knockdown of DACT2 in YES2 cells. These results indicate that the expression of cyclinB1 and CDC2 was suppressed by DACT2, and DACT2 promotes phosphorylation of CDC2 in esophageal cancer cells (Figure 5A). Above results that DACT2 induced G2/M phase arrest in human esophageal cancer cells.

### DACT2 suppresses cell migration and invasion in esophageal cancer cells

**Figure 4:**
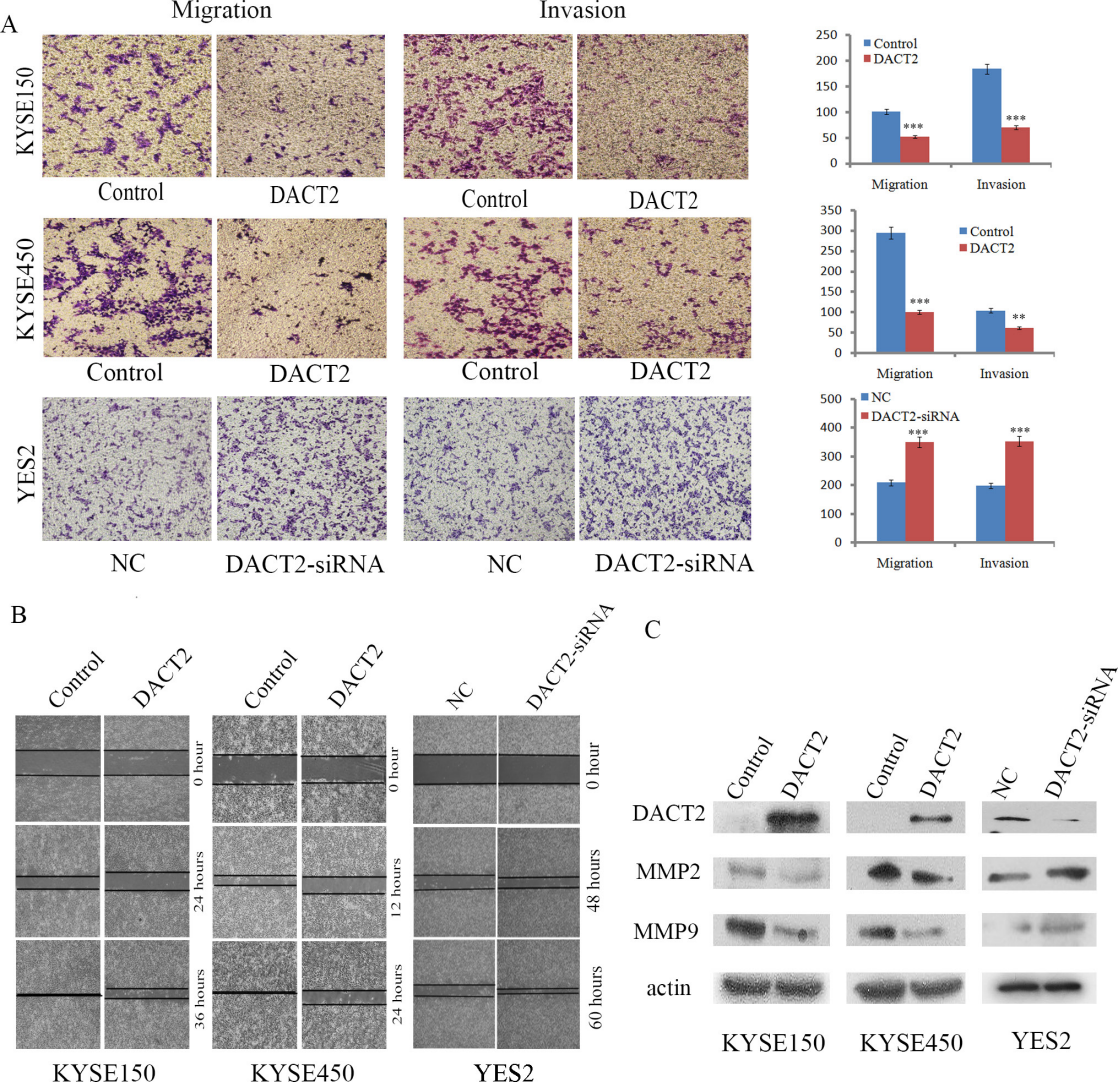
DACT2 suppresses esophageal cancer cell invasion and migration (**A**) Transwell results show cell migration and invasion in DACT2 unexpressed and re-expressed KYSE150 and KYSE450 cells, as well as migration and invasion of YES2 cells before and after knockdown of DACT2. Each experiment was repeated three times. ***P < 0.001, **P < 0.01. (**B**) Wound healing assay results in DACT2 unexpressed and re-expressed KYSE150 and KYSE450 cells, as well as the wound healing results in YES2 cells before and after knockdown of DACT2. Each experiment was repeated three times. (**C**) Western blot results in DACT2 unexpressed and re-expressed KYSE150 and KYSE450 cells, as well as YES2 cells before and after knockdown of DACT2.

To evaluate the effects of DACT2 on cell migration and invasion, the transwell and wound healing assays were employed. Under the transwell assay, the number of migratory cells was 100.67 ± 1.53 *vs*. 52.33 ± 7.51 for KYSE150 cells and 295 ± 8.72 *vs*. 99.67 ± 8.39 for KYSE450 cells before and after restoration of DACT2 expression. The cell number was reduced significantly after re-expression of DACT2 in KYSE150 and KYSE450 cells (*P* < 0.001 for both, Figure 4A). The number of migratory cells was 208.67 ± 16.29 *vs*. 350 ± 10 before and after knockdown of DACT2 in YES2 cells. The cell number was increased significantly after knockdown of DACT2 in YES2 cells (*P* < 0.001, Figure 4A). These results suggested that DACT2 suppresses esophageal cancer cell migration. The number of invasive cells was 184 ± 9.54 *vs*. 70.33 ± 3.79 for KYSE150 cells and 104 ± 15.10 *vs*. 61 ± 5.57 for KYSE450 cells before and after restoration of DACT2 expression. The invasive cell number was reduced significantly after re-expression of DACT2 in KYSE150 and KYSE450 cells (*P* < 0.001, *P* < 0.01, respectively, Figure 4A). The number of invasive cells was 198.33 ± 7.64 *vs*. 353.33 ± 12.58 before and after knockdown of DACT2 in YES2 cells. The invasive cell number was increased significantly after knockdown of DACT2 in YES2 cells (*P* < 0.001, Figure 4A). These results suggest that DACT2 suppresses esophageal cancer cell migration and invasion. The cell migration ability was evaluated by the wound healing assay as well in KYSE150 and KYSE450 cells. As shown in Figure 4B, cell migration was sharply suppressed after re-expression of DACT2 in KYSE150 and KYSE450 cells. While cell migration was promoted after knockdown of DACT2 in YES2 cells. To further explore the mechanism of DACT2 on cell migration, the expression levels of MMP2 and MMP9 were detected by western blot. As shown in Figure 4C, the expression levels of MMP2 and MMP9 were reduced after re-expression of DACT2 in KYSE150 and KYSE450 cells. MMP-2 and MMP-9 expression was increased by knocking down DACT2 in YES2 cells. Taken together, the above results demonstrate that DACT2 suppresses esophageal cancer cell invasion and migration.

### DACT2 inhibits Wnt signaling in human esophageal cancer cells

**Figure 5:**
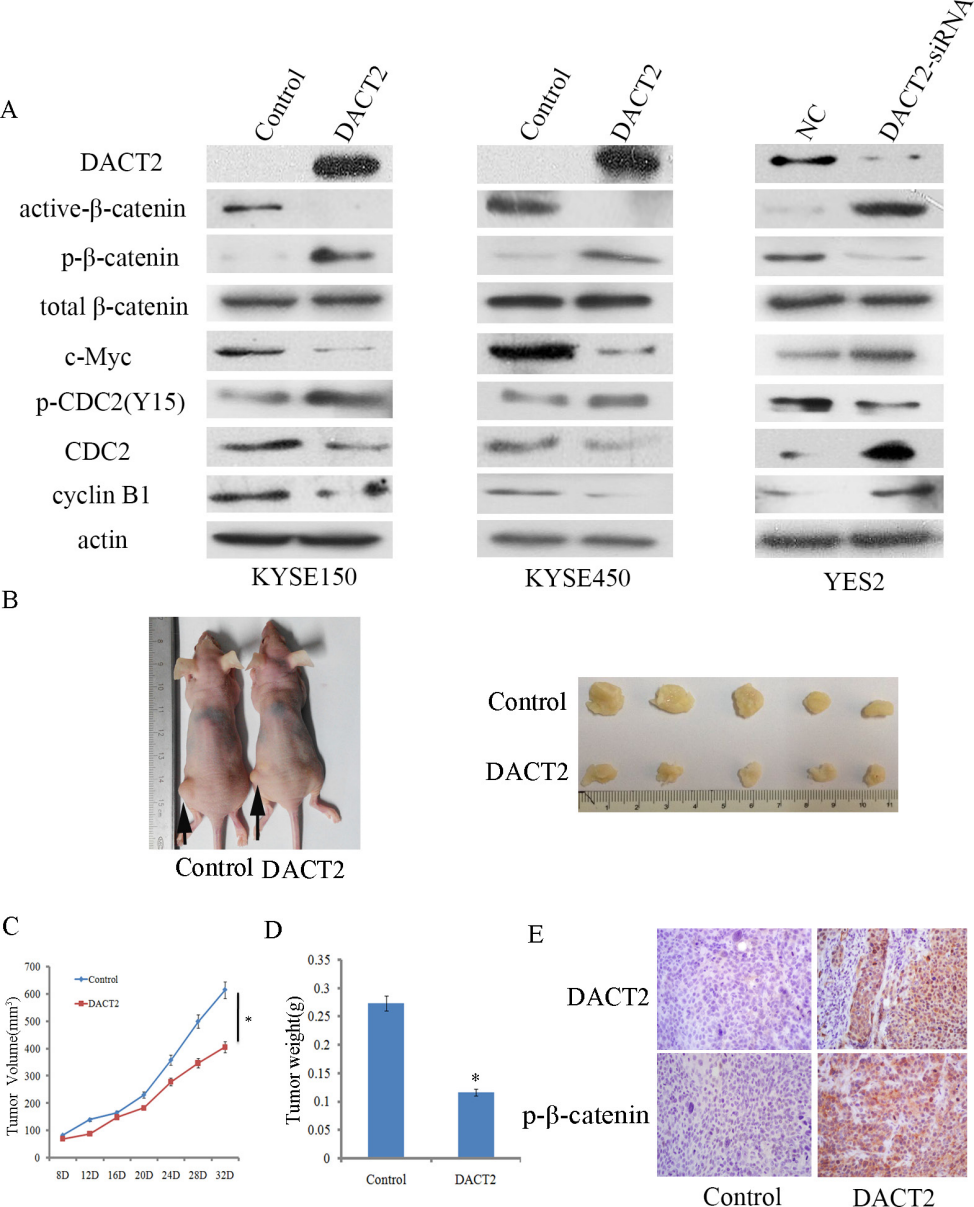
DACT2 inhibits Wnt signaling in esophageal cancer cell lines and a xenograft mouse model (**A**) The expression levels of DACT2, active-β-catenin, p-β-catenin, total β-catenin, c-Myc, p-CDC2 (Y15), CDC2 and cyclin B1 were detected by western blot in DACT2 unexpressed and re-expressed KYSE150 and KYSE450 cells. The results were validated by knocking down DACT2 in YES2 cells. (**B**) Representative results of DACT2 re-expressed and unexpressed KYSE150 cell xenograft tumors in mice. (**C**) The tumor growth curve of DACT2 re-expressed and unexpressed KYSE150 cells. *P < 0.05. (**D**) The average weight of DACT2 re-expressed and unexpressed tumors. *P < 0.05. (**E**) The expression of DACT2 (upper panels) and p-β-catenin (lower panels) in re-expressed and unexpressed KYSE150 cell xenografts by IHC. Magnification: ×400.

DACT2 is reported to suppress lung, gastric and thyroid cancers by inhibiting Wnt signaling [20, 22, 23]. The mechanisms of DACT2 in esophageal cancer were analyzed in this study. As shown in Figure 5A, the expression of total β-catenin has no changes before and after re-expression of DACT2 in KYSE150 and KYSE450 cells, or before and after knockdown of DACT2 in YES2 cells. While, the levels of active-β-catenin and c-Myc were reduced after re-expression of DACT2 in KYSE150 and KYSE450 cells, and the levels of p-β-catenin were increased after re-expression of DACT2 in KYSE150 and KYSE450 cells. The levels of active-β-catenin and c-Myc were increased and the level of p-β-catenin was reduced by knocking down DACT2 in YES2 cells. These results demonstrate that DACT2 suppresses esophageal cancer growth by inhibiting Wnt signaling.

### DACT2 suppresses human esophageal cancer cell xenograft growth

To further explore the impacts of DACT2 on esophageal cancer growth, a xenograft mouse model was established (Figure 5B). DACT2 unexpressed and re-expressed KYSE150 cells were inoculated into nude mice subcutaneously. As shown in Figure 5C, the tumor volume was 615.06 ± 164.23 mm^3^ in DACT2 unexpressed KYSE150 cell xenografts and 406.02 ± 73.87 mm^3^ in DACT2 re-expressed KYSE150 cell xenografts. The tumor volume was significantly smaller in DACT2 re-expressed KYSE150 cell line xenografts compared to DACT2 unexpressed KYSE150 cell line xenografts (*P* < 0.05, Figure 5C). The tumor weight was 0.27 ± 0.11 g and 0.12 ± 0.03 g in DACT2 unexpressed and re-expressed KYSE150 cell xenografts. The tumor weight was significantly different between these groups (*P* < 0.05, Figure 5D). To further validate the expression of DACT2 and p-β-catenin *in vivo*, the expression of DACT2 and p-β-catenin was examined by IHC staining in xenograft tumors. DACT2 was expressed in DACT2 re-expressed KYSE150 cell xenografts, and it was unexpressed in DACT2 silenced KYSE150 cell xenografts (Figure 5E, upper panels). The level of p-β-catenin increased in DACT2 re-expressed KYSE150 cell xenografts (Figure 5E, lower panels). The above results suggest that DACT2 suppresses esophageal cancer cell growth *in vivo*.

## DISCUSSION

Dapper was first identified as a Dishevelled associated antagonist of Wnt signaling in *Xenopus*, and it was demonstrated to inhibit both the canonical Wnt/β-catenin pathway and the noncanonical Wnt/c-Jun NH2-terminal kinase pathway(JNK) [18]. In zebrafish, Dapper2 (DACT2) acts as an enhancer of noncanonical Wnt signaling and has no effect on canonical Wnt/β-catenin signaling [24]. Su et al. reported that the effect of Dapper2 in mice was similar to zebrafish where it targets the TGF-β type I receptor ALK5 for degradation and has minor effects on canonical Wnt/β-catenin signaling [25]. Our previous studies demonstrated that DACT2 suppresses tumor proliferation by inhibiting canonical Wnt signaling in human hepatic, lung, thyroid and gastric cancers [20, 22, 23, 26].

Our present study mainly focused on the epigenetic regulation and function of DACT2. We found that *DACT2* is methylated in 35.7% of esophageal squamous dysplasia and 69% of human primary esophageal squamous cancer samples tested, and the expression of DACT2 is regulated by promoter region methylation. These data suggest that *DACT2* methylation may serve as an esophageal cancer early detection marker, and methylation of *DACT2* is involved in esophageal cancer development. The association of *DACT2* methylation with tumor stage suggests that methylation of *DACT2* is related to esophageal cancer progression. As *DACT2* methylation is associated to metastasis, it suggests that *DACT2* methylation may serve as a prognostic marker in ESCC. Our further investigation found that DACT2 suppresses esophageal cancer growth both *in vitro* and *in vivo*, indicating that it acts as a tumor suppressor in human esophageal cancer. *In vitro* study suggests that DACT2 induced G2/M phase arrest. To further understand the mechanism of DACT2 on cell cycle, the key regulators in G2/M checkpoint were analyzed in human esophageal cancer cells. The cyclin B1-Cdk1 (Cyclin Dependent Kinase 1, also known as cell division control protein kinase 2, CDC2) complex is a key regulator of mitotic entry [27]. A large number of proteins are phosphorylated by the cyclin B1-Cdk1 complex prior to mitotic entry, which initiates the mitotic events [28–30]. The kinase activity of Cdk1 requires phosphorylation of the threonine residue at 161 (T161) within the T-loop of Cdk1, and the phosphorylation is mediated by Cdk1-activating kinase [31]. In contrast to T161, dual phosphorylation at T14 and Y15 results in inactivation of Cdk1 [32]. Our study found that the expression levels of cyclin B1 and CDC2 were reduced, and the levels of p-CDC2 (Y15) were increased after re-expression of DACT2 in DACT2 unexpressed cells. The levels of cyclin B1 and CDC2 were increased, and the levels of p-CDC (Y15) were reduced after knockdown of DACT2 in DACT2 highly expressed YES2 cells. These results suggest that DACT2 inhibits cyclin B1 and CDC2 expression and promotes CDC2 phosphorylation at Y15. Wnt signaling plays a central role in development and carcinogenesis [33–35]. Our previous studies found DACT2 inhibits Wnt signaling in other cancers. Wnt signaling is suggested to inhibit β-catenin phosphorylation, thus inducing the accumulation of cytosolic β-catenin, which associates with the TCF/LEF (T cell factor/lymphocyte enhancer factor) family of transcription factors to activate Wnt/β-catenin-responsive genes [36–38]. The *c-Myc* proto-oncogene (*Myc*), a Wnt/β-catenin target gene, may promote the cell cycle [39, 40]. In this study, the expression of total β-catenin has no changes before and after re-expression of DACT2 in KYSE150 and KYSE450 cells, or before and after knockdown of DACT2 in YES2 cells. While, the levels of active-β-catenin and c-Myc were reduced after re-expression of DACT2 in KYSE150 and KYSE450 cells, and the levels of p-β-catenin were increased after re-expression of DACT2 in KYSE150 and KYSE450 cells. The levels of active-β-catenin and c-Myc were increased and the level of p-β-catenin was reduced by knocking down DACT2 in YES2 cells. These results further suggest that DACT2 suppresses esophageal cancer growth by inhibiting canonical Wnt signaling. Our previous findings also demonstrated that DACT2 inhibits TGF-β activity by lysosomal inhibitor-sensitive degradation of ALK4 and /or ALK5, the TGF-β receptor [41]. We conclude from these combined results that DACT2 suppresses esophageal cancer growth by inhibiting both Wnt signaling and TGF-β inhibiting. However, it remains to be elucidated which signaling pathway is primarily involved in the initiation of esophageal carcinogenesis and which signaling pathway is responsible for esophageal cancer progression and metastasis driven by DACT2.

In conclusion, *DACT2* is frequently methylated in human esophageal cancer. The expression of DACT2 is regulated by promoter region methylation. Methylation of *DACT2* is significantly associated with tumor stage and metastasis. DACT2 suppresses esophageal cancer development by inhibiting the Wnt signaling pathway.

## MATERIALS AND METHODS

### Human tissue samples and cell lines

A total of 126 cases of esophageal cancer, 42 cases of dysplasia samples and 27 cases of normal esophageal mucosa from patients without cancer were collected from the Chinese PLA General Hospital in Beijing. Snap-frozen fresh tissue samples were collected by surgery resection. All samples were collected under the guidelines approved by the institutional review board at the Chinese PLA General Hospital. Among the patient cases, 91: cases were male and 35 cases were female. The median age was 62.5 years old (range 46–87 years old). All cancer samples were classified according to the TNM staging system (AJCC2010), which included tumor stage I (*n* =4), stage II (*n* = 79), stage III (*n* = 42), and stage IV (*n* = 1). Ten esophageal cancer cell lines (KYSE30, KYSE70, KYSE140, KYSE150, KYSE450, KYSE140, TE3, TE7, TE8 and YES2) were included in this study. All esophageal cancer cell lines were previously established from primary esophageal cancer and maintained in 90% RPMI media 1640 (Invitrogen, CA, USA) supplemented with 10% fetal bovine serum. Cells were passaged 1:3 when total confluence (~10^6^ cells) was reached in a 75 cm^2^ culture flask (NEST Biotechnology, Jiangsu, China).

### 5-aza-2′-deoxycytidine treatment

Esophageal cancer cell lines were split to a low density (30% confluence) 12 hours before drug treatment. Cells were treated with 5-aza-2′-deoxycytidine (Sigma, MO, USA) at a concentration of 2 μM. Growth medium, which included 5-aza-2′-deoxycytidine at 2 μM, was exchanged every 24 hours for total of 96 hours of treatment.

### RNA isolation and semi-quantitative RT-PCR

Total RNA was isolated by Trizol reagent (Life Technology, MD, USA). Agarose gel electrophoresis and spectrophotometric analysis were used to check RNA quality and quantity. Total RNA (5 μg) was used to synthesize first strand cDNA according to the manufacturer's instructions (Invitrogen, Carlsbad, CA). The reaction mixture was diluted to 100 μl with water, and 2.5 μl of diluted cDNA mixture was added to each 25 μl PCR reaction. The DACT2 PCR primer sequences were as follows: 5′-GGC TGA GAC AAC AGG ACA TCG-3′ (F) and 5′-GAC CGT CGC TCA TCT CGT AAAA-3′ (R). Products were amplified for 35 cycles. GAPDH was amplified for 25 cycles as an internal control. The primers for GAPDH were as follows: 5′-GAC CAC AGT CCA TGC CAT CAC-3′ (F), and 5′-GTC CAC CAC CCT GTT GCT GTA-3′ (R). The amplified PCR products were examined by 1.5% agarose gels.

### Bisulfite modification, methylation-specific PCR and bisulfite sequencing

Genomic DNA was extracted by the proteinase K method. The bisulfite modification assay was performed as previously described [42]. MSP primers were designed according to genomic sequences around the transcription start sites (TSS) and synthesized (BGI, Beijing, China) to detect unmethylated (U) and methylated (M) alleles. MSP primers were as follows: 5′-GCG CGT GTA GAT TTC GTT TTT CGC-3′ (MF) and 5′-AAC CCC ACG AAC GAC GCCG-3′ (MR); 5′-TTG GGG TGT GTG TAG ATT TTG TTT TTT GT-3′ (UF) and 5′-CCC AAA CCC CAC AAA CAA CAC CA-3′ (UR). The expected sizes of unmethylated and methylated products were 161 bp and 152 bp, respectively. Bisulfite-treated DNA was also amplified using bisulfite sequencing (BSSQ) primers that included the MSP region. The sequencing primers were as follows: 5′-GGG GGA GGT YGY GGT GAT TT-3′ (F) and 5′-ACC TAC RAC RAT CCC AAC CC-3′ (R). Bisulfite sequencing was performed as previously described [43].

### Immunohistochemistry

Immunohistochemistry (IHC) was performed in primary esophageal cancer and paired adjacent tissue samples. DACT2 antibody was diluted to 1/400 dilution (Cat: TA306668, OriGene Tech., MD, USA). The staining intensity and extent of the stained area were scored using the German semi-quantitative scoring system. The staining intensity of DACT2 expression was quantified as follows: no staining = 0; weak staining = 1; moderate staining = 2; strong staining = 3. The extent of DACT2 expression was quantified as follows: 0% = 0, 1–24% = 1, 25–49% = 2, 50–74% = 3, 75–100% = 4 [44, 45]. The final immune-reactive score (0 to 12) was determined by multiplying the intensity score to the extent of stained cells score.

### Construction of lentiviral DACT2 expression vectors and selection of stable expression cells

The human full length *DACT2* cDNA (GenBank accession number NM_214462) was cloned into the pLenti6-GFP vector [22]. Primers were as follows: 5′-TGA TCA ATG TGG ACG CCG GGC-3′ (F) and 5′-GTC GAC TCA CAC CAT GGT CAT GAC-3′(R). The HEK-293T cell line was maintained in 90% DMEM (Invitrogen, CA, USA) supplemented with 10% fetal bovine serum. DACT2 expressing Lentiviral vector was transfected into HEK-293T cells (5 × 10^6^ per 100 mm dish) using Lipofectamine 2000 Reagent (Invitrogen, CA, USA) at a ratio of 1:3 (DNA mass : Lipo mass). Viral supernatant was collected and filtered after 48 hours. KYSE150 and KYSE450 cell lines were then infected with viral supernatant. Cells stably expressing DACT2 were selected with Blasticidin (Life Technologies, MD, USA) at concentrations of 0.2 μg/ml (KYSE150) and 0.4 μg/ml (KYSE450) for 2 weeks.

### Cell viability assay

DACT2 stably expressed and unexpressed KYSE150 and KYSE450 cells were plated into 96-well plates at a density of 3 × 10^3^ cells/well. 5 × 10^3^ cells were plated into 96-well plates before and after knockdown of DACT2 in YES2 cells. The cell viability was measured by the MTT assay at 0, 24, 48 and 72 h (KeyGEN Biotech, Nanjing, China). Absorbance was measured on a microplate reader (Thermo Multiskan MK3, MA, USA) at a wave length of 490 nm. The results were plotted as means ± SD.

### Colony formation assay

DACT2 stably expressed and unexpressed KYSE150 and KYSE450 cell lines were seeded in 6-well plates at a density of 1000 cells per well. YES2 cells before and after knockdown of DACT2 were seeded in 6-well plates at a density of 1000 cells per well. Growth medium, which included blasticidin at 0.2 μg/ml (KYSE150) or 0.4 μg/ml (KYSE450), was exchanged every 24 hours. Cells were fixed with 75% ethanol for 30 min and stained with 0.2% crystal violet after 14 days. The number of clones was then counted. Each experiment was repeated three times.

### Flow cytometry

DACT2 stably re-expressed and unexpressed KYSE150 and KYSE450 cells were starved 12 hours for synchronization, and cells were re-stimulated with 10% FBS for 24 hours. Cells were fixed with 70% ethanol and treated using the Cell Cycle Detection Kit (KeyGen Biotech, Nanjing, China). Cells were then detected using a FACS Caliber flow cytometer (BD Biosciences, CA, USA). YES2 cells with or without knockdown of DACT2 were analyzed the cell cycle as well. Cells phase distribution was analyzed using the Modfit software (Verity Software House, ME, USA).

### Transwell assay

Migration: 1 × 10^5^ DACT2 unexpressed and re- expressed KYSE150 and KYSE450 cells, were suspended in 200 μl serum-free RPMI 1640 media and added to the upper chamber of 8.0 μm pore size transwell apparatus (COSTAR transwell, Corning Incorporated, MA, USA). Cells that migrated to the lower surface of the membrane were stained with crystal violet and counted in three independent high-power fields (×100) after incubating for 20 hours. 8 × 10^4^ YES2 cells before and after knockdown of DACT2 were added to the upper chamber of 8.0 μm pore size transwell apparatus. Cells were migrated to the lower surface of the membrane after incubating for 36 hours.

Invasion: the top chamber was coated with a layer of extracellular matrix. Cells (2 × 10^5^) were seeded to the upper chamber of a transwell apparatus coated with Matrigel (BD Biosciences, CA, USA) and incubated for 36 hours. Cells that invaded to the lower membrane surface were stained with crystal violet and counted in three independent high-power fields (×100). 1 × 10^5^ YES2 cells before and after knockdown of DACT2 were added to the upper chamber of a transwell apparatus coated with Matrigel. Cells were invaded to the lower membrane surface after incubating for 48 hours.

### Wound healing assay

Linear scratch wounds were created with a pipette tip in a confluent monolayer of DACT2 unexpressed and re-expressed KYSE150 and KYSE450 cells in 6-well plates, linear scratch wounds were also created in YES2 cells growing 6-well plates before and after knockdown of DACT2. Cells were grown in FBS-free medium for inhibition of cell proliferation.

### SiRNA knockdown technique

The selected siRNAs targeting DACT2 and RNAi negative control duplex were used in this study. The sequences were as follows: siRNA duplex (sense: 5′-GCC UGU GUC UAC AGG UGA UTT-3′; antisense: 5′-AUC ACC UGU AGA CAC AGG CTT-3′); RNAi negative control duplex (sense: 5′-UUC UCC GAA CGU GUC ACG UTT-3′; antisense: 5′-ACG UGA CAC GUU CGG AGA ATT-3′). RNAi oligonucleotide or RNAi negative control duplex (Gene Pharma Co. Shanghai, China) were transfected into DACT2 highly expressed YES2 cells.

### Protein preparation and western blot

Protein samples from unexpressed and stably re-expressed KYSE150 and KYSE450 cells were collected and western blots were performed as described previously [44]. Antibodies were diluted according to the manufacturer's instructions. Primary antibodies included DACT2 (Cat: TA306668, OriGene Tech, MD, USA), c-Myc (Cat: 10828-1-AP, proteintech, IL, USA), cyclin B1 (Cat: 55004-1-AP, proteintech, IL, USA), CDC2 (Cat: 19532-1-AP, proteintech, IL, USA), p-CDC2 (Y15) (Cat: #9111, Cell Signaling Tech, MA, USA), MMP2 (Cat: BS1236, Bioworld Tech, MN, USA), MMP9 (Cat: BS1241, Bioworld Tech, MN, USA), Anti-Active-β-Catenin, clone8E7 (Cat: #05-665, Millipore, CA, USA), p-β-catenin (S37) (Cat: BS4739, Bioworld Tech, MN, USA), β-catenin (D10A8) XP^®^ (Cat: #8480, Cell Signaling Tech, MA, USA) and β-actin (Cat: AF0003, Beyotime Biotech, Jiangsu, China).

### The effect of DACT2 on KYSE150 cell xenograft

Stably transfected KYSE150 cell line with pLenti6-GFP vector or pLenti6-DACT2 vector (4 × 10^6^ cells in 0.15 ml phosphate-buffered saline) were injected subcutaneously into the dorsal left side of 4–week-old male Balb/c nude mice (*n* = 5). Tumor volume was measured every 4 days for 24 days starting 8 days after implantation. Tumor volume was calculated according to the formula: V = L × W^2^/2, where V represents volume (mm^3^), L represents biggest diameter (mm), and W represents smallest diameter (mm). All procedures were approved by the Animal Ethics Committee of the Chinese PLA General Hospital.

### Statistical analysis

SPSS 17.0 software (IBM, NY, USA) software was applied using χ^2^ test for independent dichotomous variables. All data were presented as means ± standard deviation (SD) of at least three independent experiments and analyzed using the student's *t* test. Results were reported to be statistically significant at *P* < 0.05(*).
